# Actinobacterial Degradation of 2-Hydroxyisobutyric Acid Proceeds via Acetone and Formyl-CoA by Employing a Thiamine-Dependent Lyase Reaction

**DOI:** 10.3389/fmicb.2020.00691

**Published:** 2020-04-15

**Authors:** Thore Rohwerder, Maria-Teresa Rohde, Nico Jehmlich, Jessica Purswani

**Affiliations:** ^1^Department of Environmental Microbiology, Helmholtz Centre for Environmental Research, Leipzig, Germany; ^2^Institut für Chemie – Biophysikalische Chemie, Martin-Luther-Universität Halle-Wittenberg, Halle (Saale), Germany; ^3^Department of Molecular Systems Biology, Helmholtz Centre for Environmental Research, Leipzig, Germany; ^4^Institute of Water Research, University of Granada, Granada, Spain

**Keywords:** degradation pathway, isobutene, *tert*-butyl alcohol, fuel oxygenate, *Mycolicibacterium*, 2-hydroxyacyl-CoA lyase, acyloin condensation

## Abstract

The tertiary branched short-chain 2-hydroxyisobutyric acid (2-HIBA) has been associated with several metabolic diseases and lysine 2-hydroxyisobutyrylation seems to be a common eukaryotic as well as prokaryotic post-translational modification in proteins. In contrast, the underlying 2-HIBA metabolism has thus far only been detected in a few microorganisms, such as the betaproteobacterium *Aquincola tertiaricarbonis* L108 and the *Bacillus* group bacterium *Kyrpidia tusciae* DSM 2912. In these strains, 2-HIBA can be specifically activated to the corresponding CoA thioester by the 2-HIBA-CoA ligase (HCL) and is then isomerized to 3-hydroxybutyryl-CoA in a reversible and B_12_-dependent mutase reaction. Here, we demonstrate that the actinobacterial strain *Actinomycetospora chiangmaiensis* DSM 45062 degrades 2-HIBA and also its precursor 2-methylpropane-1,2-diol via acetone and formic acid by employing a thiamine pyrophosphate-dependent lyase. The corresponding gene is located directly upstream of *hcl*, which has previously been found only in operonic association with the 2-hydroxyisobutyryl-CoA mutase genes in other bacteria. Heterologous expression of the lyase gene from DSM 45062 in *E. coli* established a 2-hydroxyisobutyryl-CoA lyase activity in the latter. In line with this, analysis of the DSM 45062 proteome reveals a strong induction of the lyase-HCL gene cluster on 2-HIBA. Acetone is likely degraded via hydroxylation to acetol catalyzed by a MimABCD-related binuclear iron monooxygenase and formic acid appears to be oxidized to CO_2_ by selenium-dependent dehydrogenases. The presence of the lyase-HCL gene cluster in isoprene-degrading *Rhodococcus* strains and *Pseudonocardia* associated with tropical leafcutter ant species points to a role in degradation of biogenic short-chain ketones and highly branched organic compounds.

## Introduction

**FIGURE 1:**
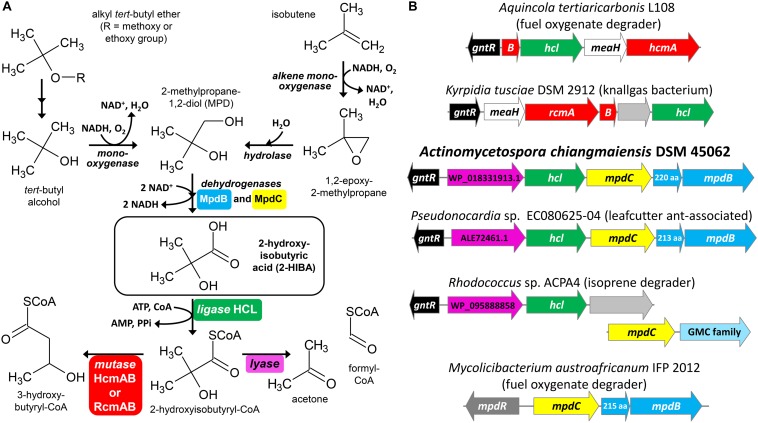
Bacterial conversion of *tert*-butyl compounds. **(A)** Proposed pathways for the degradation of fuel oxygenate ethers and isobutene via MPD and 2-HIBA. **(B)** Examples for gene clusters with *mpdBC* and/or *hcl* genes encoding the enzymes for the two-step oxidation of MPD to 2-HIBA and its activation to 2-hydroxyisobutyryl-CoA, respectively. The magenta colored genes from strain DSM 45062 and in the clusters of strains EC080625-04 and ACPA4 correspond to proteins WP_018331913.1, ALE72461.1 and WP_095888858.1, respectively, which likely function as TPP-dependent 2-hydroxyacyl-CoA lyases, for example, for the cleavage of 2-hydroxyisobutyryl-CoA to acetone and formyl-CoA. GMC family, glucose-methanol-choline oxidoreductase family.

2-Hydroxyisobutyric acid (2-HIBA) is a short-chain carboxylic acid bearing a tertiary hydroxyl group (Figure 1A). It has been viewed at as a mainly xenobiotic compound associated with the degradation of the structurally related *tert*-butyl moieties of the fuel oxygenates methyl and ethyl *tert*-butyl ether (Rohwerder et al., 2006). Additionally, 2-HIBA and its methyl ester can be formed as intermediates in industrial processes for the production of acrylic glass (Rohwerder and Müller, 2010), i.e., poly(methyl methacrylate). In the last years, however, 2-HIBA has been proposed as biomarker for several metabolic diseases, such as diabetes mellitus and adiposity (Li et al., 2009; Elliott et al., 2015), and increased titers are also associated with alcohol abuse (Irwin et al., 2018). Furthermore, it has recently been discovered that lysine 2-hydroxyisobutyrylation is an important post-translational modification of histones and other proteins (Dai et al., 2014; Huang et al., 2017). All this indicates now that 2-HIBA and its metabolism is much more widespread in both prokaryotes and eukaryotes than previously assumed. Nevertheless, enzymes specifically catalyzing the synthesis and degradation of 2-HIBA and its CoA thioester 2-hydroxyisobutyryl-CoA have thus far only been characterized in a few bacterial strains.

Under oxic conditions, alkyl *tert*-butyl ethers are degraded via *tert*-butyl alcohol which is further oxidized to 2-methylpropane-1,2-diol (MPD) and 2-HIBA (Figure 1A) (Zahn et al., 2019). Accordingly, the MPD and 2-HIBA pathways have been studied first in aerobic fuel oxygenate degraders, such as the betaproteobacterium *Aquincola tertiaricarbonis* L108 (Schäfer et al., 2011, 2012; Schuster et al., 2012) and the actinobacterium *Mycolicibacterium austroafricanum* IFP 2012 (Francois et al., 2002, 2003; Lopes Ferreira et al., 2006a). In the latter, the dehydrogenases MpdB and MpdC catalyze the oxidation of MPD and 2-hydroxyisobutyraldehyde, respectively, to 2-HIBA. The subsequent steps might proceed via isopropanol and acetone, but have not yet been elucidated in strain IFP 2012 or other actinobacteria (Lopes Ferreira et al., 2006b). In contrast, strain *A. tertiaricarbonis* L108 CoA activates 2-HIBA employing the specific 2-HIBA-CoA ligase (HCL) (Zahn et al., 2019). The CoA thioester is then isomerized by the B_12_-dependent acyl-CoA mutase HcmAB to (*S*)-3-hydroxybutyryl-CoA (Yaneva et al., 2012; Kurteva-Yaneva et al., 2015). The mutase route has also been found in the Gram-positive bacterium *Kyrpidia tusciae* DSM 2912 (Weichler et al., 2015). Interestingly, here the mutase RcmAB, which is only distantly related to HcmAB, catalyzes the isomerization reaction, preferentially forming (*R*)-3-hydroxybutyryl-CoA. And *Bacillus massiliosenegalensis* JC6 even possesses both 2-hydroxyisobutyryl-CoA mutase forms (Rohde et al., 2017). The latter and also other bacterial strains bearing the mutase-HCL gene cluster (Figure 1B) have not been associated with the degradation of fuel oxygenates or other xenobiotics, but mostly seem to be isolated from pristine environments, such as geothermal solfatara ponds, marine dinoflagellates, agricultural soil and root nodules from *Fabaceae* species (Weichler et al., 2015; Zahn et al., 2019). This might be indicative of a natural source of 2-HIBA or a related carboxylic acid not yet identified. In this connection, it has been shown that isobutene degradation in strain *Mycobacterium* sp. ELW1 proceeds via the corresponding epoxide, MPD and 2-HIBA (Kottegoda et al., 2015), likely employing a plasmid-borne (pELW1-1, NZ_CP032156.1) alkene monooxygenase, an epoxide hydrolase and MpdBC-related dehydrogenase steps (Figure 1A).

As mentioned above, 2-HIBA might not be degraded exclusively via the mutase pathway and it has been speculated whether an alternative route could lead to isopropanol or acetone via a cleavage reaction, for example, a decarboxylation of the free carboxylic acid in the actinobacterial strains IFP 2012 and *Mycolicibacterium vaccae* JOB5 (Lopes Ferreira et al., 2006b). However, for these reactions neither the relevant metabolites nor the enzyme activities have been unambiguously demonstrated. Here, we investigate MPD and 2-HIBA metabolism in the actinobacterial strain *Actinomycetospora chiangmaiensis* DSM 45062, which has previously been isolated from tropical rainforest soil, Chiang Mai, Thailand (Jiang et al., 2008). Inspection of the genome sequence (NZ_ARBI00000000.1) revealed the absence of genes encoding 2-hydroxyisobutyryl-CoA mutases. However, *hcl* is present in a gene cluster comprising *mpdB* and *mpdC* as well as a gene predicted to encode a thiamine pyrophosphate (TPP)-dependent enzyme. This suggests a metabolic sequence from MPD via 2-HIBA to 2-hydroxyisobutyryl-CoA which might then be cleaved to acetone and formyl-CoA employing a TPP-dependent lyase reaction (Figure 1A). The postulated pathway is corroborated by detection of the corresponding metabolites and by proteome analysis showing a strong induction of the lyase-HCL gene cluster in strain *A. chiangmaiensis* DSM 45062 when grown on 2-HIBA. In addition, enzymatic acetone formation from 2-hydroxyisobutyryl-CoA was demonstrated in the wild-type strain and after heterologous expression of the lyase gene in *E. coli*.

## Results

### Organization of the Lyase-HCL Gene Cluster in *A. chiangmaiensis* DSM 45062

In the fuel oxygenate-degrading *A. tertiaricarbonis* L108 and *M. petroleiphilum* PM1 as well as in many other bacterial strains (Yaneva et al., 2012; Weichler et al., 2015), the gene encoding the specific ligase for the CoA-activation of 2-HIBA, *hcl*, is in operon-like organization with the 2-hydroxyisobutyryl-CoA mutase genes, *hcmAB* or *rcmAB*, and *meaH* encoding a mutase-associated G-protein chaperone. In addition, upstream on the same or the complement strand, a gene for a putative transcriptional regulator of the GntR family is usually present (Figure 1B). However, a BLASTP search (NCBI non-redundant protein sequences) revealed that *hcl* also occurs in gene clusters lacking the mutase genes. In strain *A. chiangmaiensis* DSM 45062, for example, *hcl* is associated with two genes annotated as *mpdB* and *mpdC* (Figure 1B). The corresponding proteins MpdB and MpdC are closely related to the alcohol and aldehyde dehydrogenases, respectively, from *M. austroafricanum* IFP 2012 (76 and 72% sequence identity, respectively, at 99% query coverage) which have previously been demonstrated to be involved in the two-step oxidation of the methyl *tert*-butyl ether metabolite MPD to 2-HIBA via 2-hydroxyisobutyraldehyde (Lopes Ferreira et al., 2006a). This indicates that strain *A. chiangmaiensis* DSM 45062 might be capable of converting MPD to 2-hydroxyisobutyryl-CoA. Further degradation, however, does obviously not employ a B_12_-dependent mutase, but seems to proceed via an alternative route. Interestingly, the first ORF in the *hcl* gene cluster, NCBI RefSeq WP_018331913.1, is predicted to encode a protein of 590 aa length belonging to the TPP-requiring decarboxylase superfamily (Vogel and Pleiss, 2014) and might function as a 2-hydroxyacyl-CoA lyase. The human peroxisomal 2-hydroxyphytanoyl-CoA lyase HACL1 (NP_036392.2), for example, catalyzes the decomposition of 2-hydroxyphytanoyl-CoA (2-hydroxy-3,7,11,15-tetramethylhexadecanoyl-CoA) to the aldehyde pristanal (2,6,10,14-tetramethylpentadecanal) and formyl-CoA (Foulon et al., 1999, 2005). In addition, it has recently been demonstrated that HACL1 and related prokaryotic TPP-dependent enzymes can even catalyze the reversible acyloin condensation of formyl-CoA with short- and medium-chain carbonyl compounds (Chou et al., 2019; Burgener et al., 2020). Although these 2-hydroxyacyl-CoA lyases and the putative lyase from DSM 45062 are only distantly related (<30% sequence identity at about 90% query coverage), this provisional, only sequence-based functional assignment of WP_018331913.1 let us speculate about a mutase-independent pathway for the degradation of MPD and 2-HIBA proceeding via the decomposition of 2-hydroxyisobutyryl-CoA to acetone and formyl-CoA (Figure 1A).

### Degradation of MPD via 2-HIBA, Acetone and Formic Acid

**FIGURE 2:**
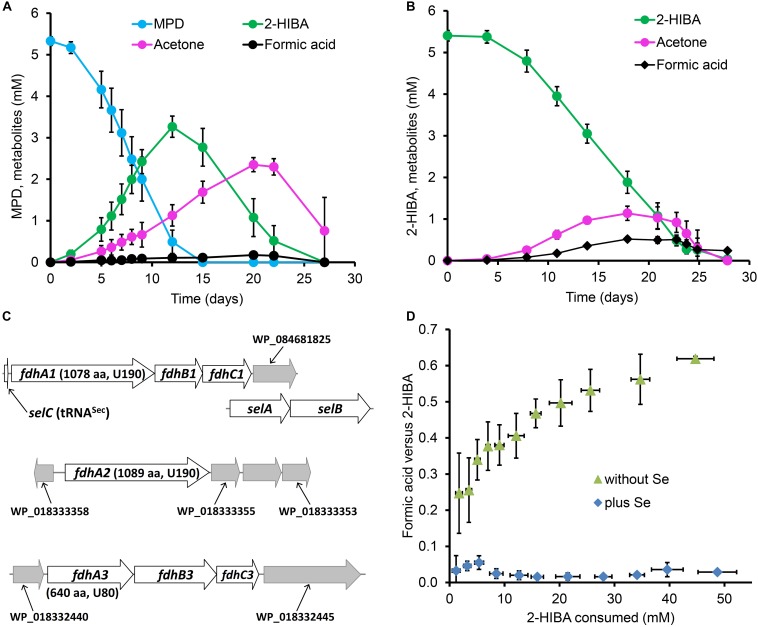
Metabolites of *tert*-butyl compound conversion and selenium dependence of formic acid removal in strain *A. chiangmaiensis* DSM 45062. Cultures degrading **(A)** MPD and **(B)** 2-HIBA as main carbon source when incubated in mineral salt medium not supplemented with selenium. **(C)** Gene clusters encoding selenocysteine-containing FDHs and associated genes. Predicted position of the selenocysteine in the corresponding FdhA subunits and the protein length are indicated. **(D)** Molar ratio of formic acid formation versus 2-HIBA consumption in fed-batch cultures with 2-HIBA as main carbon source incubated in mineral salt medium with or without selenium supplementation.

In order to elucidate the 2-HIBA pathway in strain DSM 45062, cultures were incubated in a mineral salt medium supplemented either with 5.5 mM MPD or 5.5 mM 2-HIBA as main carbon sources. On both substrates, growth was poor and biomass was produced exclusively as small cell aggregates (up to about 3 mm, Supplementary Figure S1). Based on dry weight increase, roughly 0.2 g biomass were formed per g of MPD and 2-HIBA consumed. Degradation of MPD was accompanied by temporary formation of up to 3.3 and 2.4 mM 2-HIBA and acetone, respectively (Figure 2A). Besides, low amounts of formic acid (<0.2 mM) could be detected. Likewise, 2-HIBA was slowly degraded via acetone and formic acid (Figure 2B), confirming the postulated cleavage of the C4 carboxylic acid into C3 and C1 compounds (Figure 1A).

### Selenium Dependence of Formic Acid Degradation

From the DSM 45062 genome, three multicomponent formate dehydrogenase (FDH) systems with alpha subunits bearing a selenocysteine residue within the conserved domain for binding the molybdenum/tungsten of the metallopterin cofactor (Hartmann et al., 2015) can be deduced (Figure 2C, Supplementary Figures S2–S4), whereas a selenium-independent formic acid oxidation is not encoded. Accordingly, substantial accumulation of formic acid was observed in prolonged fed-batch experiments when enabling degradation of up to 50 mM 2-HIBA in medium not supplemented with selenite (Figure 2D). However, this accumulation was not stoichiometric but only reached a molar ratio of about 0.6. Nevertheless, when supplementing the medium with selenite (0.11 μM), formic acid formation was almost negligible (Figure 2D) which is clearly corroborative of a selenocysteine-containing FDH step involved in degradation of the C1 cleavage product from 2-HIBA. Supplementation of the medium with tungsten (0.12 μM as sodium tungstate) did not show any effect on formic acid production from 2-HIBA, indicating that the active FDHs in strain DSM 45062 are binding the molybdenum-containing cofactor (Gladyshev et al., 1994). In contrast to the 2-HIBA cultures, fed-batch growth on acetone as main carbon source did not require supplementation with selenium, in line with a degradation pathway not proceeding via formic acid formation.

### Proteome of Acetone- and 2-HIBA-Grown Cells

**FIGURE 3:**
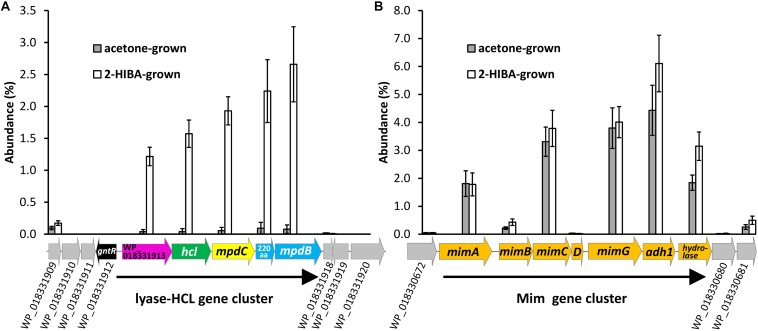
Induction of **(A)** the lyase-HCL and **(B)** the Mim gene clusters in fed-batch cultures of *A. chiangmaiensis* DSM 45062 grown on 2-HIBA or acetone in mineral salt medium supplemented with selenium. For comparison, abundance of proteins encoded by genes directly up- and downstream of the clusters is shown as well.

**FIGURE 4:**
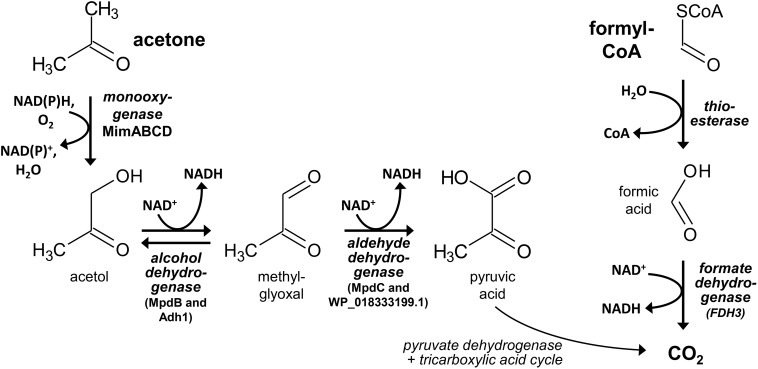
Proposed reaction steps for the degradation of the 2-hydroxyisobutyryl-CoA cleavage products acetone and formyl-CoA in strain *A. chiangmaiensis* DSM 45062. Likely, hydroxylation of acetone and the subsequent acetol oxidation to methylglyoxal are catalyzed by the Mim cluster diiron monooxygenase and alcohol dehydrogenase Adh1 (WP_018330678.1), respectively. Only in 2-HIBA-grown cells, alcohol oxidation may also involve MpdB and methylglyoxal could be oxidized by MpdC. By contrast, the aldehyde oxidation is likely taken over by dehydrogenase WP_018333199.1 when cells are incubated on acetone. Formyl-CoA may be hydrolyzed to formic acid, which can be oxidized by 2-HIBA-induced cytoplasmic NAD^+^-dependent formate dehydrogenase FDH3.

For identifying the key enzyme(s) catalyzing the 2-HIBA cleavage reaction, the proteome of DSM 45062 cells from fed-batch acetone and 2-HIBA cultures (both supplemented with selenite) was compared. Interestingly, expression of the complete lyase-HCL cluster is strongly induced on 2-HIBA resulting in an abundance of the corresponding gene products between 1.2 and 2.7% relative abundance of the whole proteome (Figure 3A and Table 1). In stark contrast, these proteins are almost absent in cells grown on acetone. Cells from both culture variants, however, show strong induction of a gene cluster encoding components of a binuclear iron monooxygenase (Figure 3B and Table 1) closely related to the MimABCD system involved in acetone hydroxylation in strains *Mycolicibacterium goodii* 12523 and *Mycolicibacterium smegmatis* mc^2^155 (Furuya et al., 2011, 2015). Hence, also in strain DSM 45062 acetone is likely hydroxylated to acetol (1-hydroxy-2-propanone) and might be subsequently oxidized to methylglyoxal and pyruvate (Figure 4). Enzyme candidates for catalyzing these alcohol and aldehyde oxidations are the putative isopropanol dehydrogenase of the Mim cluster (Adh1, WP_018330678.1) and a 507-aa aldehyde dehydrogenase (WP_018333199.1), respectively. The latter is only induced on acetone (abundance 5.2 ± 1.1%) and might be replaced by MpdC in 2-HIBA-grown cells. Besides, several other putative alcohol and aldehyde dehydrogenases are substantially induced on acetone, 2-HIBA or both substrates and might play a role in acetone degradation as well, for example, the Adh1-related WP_018331648.1 (Table 1). Among these enzymes are also alcohol dehydrogenases (WP_018332348.1 mainly induced on acetone at an abundance of about 4% and WP_018334013.1 on both substrates at values between 0.4 and 0.8%) that are predicted to use 4-nitroso-*N,N*-dimethylaniline as artificial and mycofactocin as endogenous cofactor, as has recently been elucidated for a related dehydrogenase involved in ethanol oxidation (Krishnamoorthy et al., 2019). Remarkably, among the most abundant proteins is also a putative catalase/peroxidase (WP_033414714.1 with an abundance between 1.6 and 4.2%) which is indicative of high stress by reactive oxygen species, possibly produced in the course of uncoupled MimABCD activity. As expected from the cultivation experiments, FDH gene clusters as well as the machinery for selenocysteine synthesis and its incorporation into proteins (SelD, SelA, and SelB) were expressed on 2-HIBA. However, the corresponding proteins were only detected at low abundance, with values between 0.001 and 0.02% (Table 1). Compared to acetone-grown cells, the fold-change in FdhA1, FdhB1 and the three FDH3 subunits was significant, albeit showing only maximal up-regulation of about threefold.

**TABLE 1:** Proteome analysis of *A. chiangmaiensis* DSM 45062.

**Predicted properties and functions**	**NCBI ID**	**Gene cluster**	**Length (aa)**	**Abundance (%)**	**Fold-change**
GntR family transcriptional regulator (GntR)	WP_018331912.1		241	n.d.	n.a.
2-Hydroxyisobutyryl-CoA lyase, TPP-dependent	WP_018331913.1	Lyase-HCL	590	1.2 ± 0.1	29
2-HIBA-CoA ligase (HCL)	WP_018331914.1	Lyase-HCL	465	1.6 ± 0.2	39
2-Hydroxyisobutyraldehyde dehydrogenase (MpdC), reduces NAD(P)^+^	WP_018331915.1	Lyase-HCL	501	1.9 ± 0.2	34
Small subunit of MPD dehydrogenase	WP_018331916.1	Lyase-HCL	220	2.2 ± 0.5	23
Large subunit of MPD dehydrogenase (MpdB), reduces NAD(P)^+^	WP_018331917.1	Lyase-HCL	550	2.7 ± 0.6	33
Monooxygenase large subunit (MimA)	WP_085942306.1	Mim	550	1.8 ± 0.4	n.s.
Monooxygenase reductase (MimB), oxidizes NAD(P)H	WP_018330674.1	Mim	348	0.4 ± 0.1	1.9
Monooxygenase small subunit (MimC)	WP_018330675.1	Mim	395	3.8 ± 0.7	n.s.
Monooxygenase coupling protein (MimD)	WP_018330676.1	Mim	131	0.02 ± 0.01	0.6
Acetone monooxygenase, GroEL-like protein (MimG), maturation of MimAC	WP_026204221.1	Mim	561	4.0 ± 0.6	n.s.
Zn-dependent alcohol dehydrogenase (Adh1), reduces NAD(P)^+^	WP_018330678.1	Mim	342	6.1 ± 1.0	1.3
Amidohydrolase-like protein	WP_018330679.1	Mim	349	3.2 ± 0.5	1.6
Zn-dependent alcohol dehydrogenase, reduces NAD(P)^+^	WP_018331648.1		342	3.3 ± 1.1	n.s.
Alpha subunit of membrane-bound FDH (FdhA1)	n.a.	FDH1	1078	0.011 ± 0.003	1.9
FDH beta subunit (FdhB1)	WP_018332710.1	FDH1	362	0.012 ± 0.007	Only on 2-HIBA
FDH gamma subunit (FdhC1)	WP_018332711.1	FDH1	366	0.002 ± 0.003	n.s.
Permease (transmembrane protein)	WP_084681825.1	FDH1	322	n.d.	n.a.
Selenocysteine synthase (SelA)	WP_018332713.1	FDH1	459	0.001 ± 0.001	0.4
Selenocysteine-specific elongation factor (SelB)	WP_018332714.1	FDH1	597	0.001 ± 0.002	n.s.
Selenide, water dikinase (SelD), donor of selenophosphate	WP_018332294.1		334	0.02 ± 0.01	n.s.
Alpha subunit of membrane-bound FDH (FdhA2)	n.a.	FDH2	1089	0.002 ± 0.003	n.s.
Alpha subunit of cytoplasmic FDH (FdhA3)	WP_084681743.1	FDH3	640	0.012 ± 0.002	3.1
FDH beta subunit (FdhB3)	WP_018332443.1	FDH3	614	0.022 ± 0.005	2.8
FDH gamma subunit (FdhC3)	WP_018332444.1	FDH3	316	0.017 ± 0.005	2.2
Formate dehydrogenase accessory sulfurtransferase (FdhD)	WP_026205059.1		280	0.003 ± 0.002	n.s.

*Cells were grown on either 2-HIBA or acetone as main carbon source. Abundance in 2-HIBA-grown cells and fold-change (2-HIBA versus acetone) of proteins probably involved in 2-HIBA degradation; n.a., not applicable; n.d., not detected (neither on 2-HIBA nor on acetone); n.s., not significant.*

### WP_018331913.1-Dependent 2-Hydroxyisobutyryl- and 2-Hydroxy-2-Methylbutyryl-CoA Lyase Activity

**FIGURE 5:**
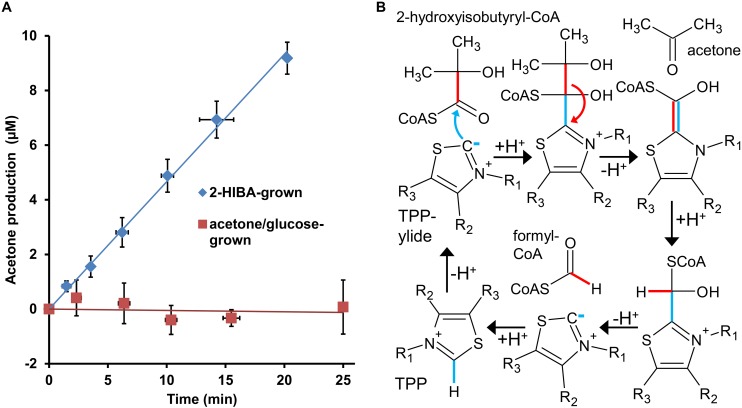
2-Hydroxyisobutyryl-CoA lyase activity in strain *A. chiangmaiensis* DSM 45062. **(A)** TPP-dependent acetone formation from 2-hydroxyisobutyryl-CoA in crude extracts of cells grown on main carbon sources as indicated. The lyase activity obtained from 2-HIBA-grown cells amounts to 2.9 ± 0.1 nmol min^–1^ mg protein^–1^, while it is absent when the strain was incubated on acetone or glucose. **(B)** Mechanism for the reverse acyloin condensation of 2-hydroxyisobutyryl-CoA proposed to be catalyzed by the lyase from strain DSM 45062. The TPP-dependent reaction starts by deprotonation of the cofactor to yield the active ylide form. For simplicity, only the thiazole ring of TPP is shown.

In line with the proteome results demonstrating significant expression of the predicted lyase gene, corresponding to WP_018331913.1, only in 2-HIBA-grown cells, crude extracts from the latter clearly show acetone formation at a rate of about 3 nmol min^–1^ mg protein^–1^ when incubated with 2-hydroxyisobutyryl-CoA, while this activity was not detected in cell-free preparations derived from acetone and glucose cultures (Figure 5A). Moreover, the 2-hydroxyisobutyryl-CoA lyase activity is stimulated by supplementation with TPP (optimal concentration at about 200 μM), in line with a cleavage mechanism dependent on this cofactor (Figure 5B). Similar activity of about 6 nmol min^–1^ mg^–1^ was established in crude extracts from *E. coli* cells heterologously expressing the lyase gene, confirming the role of WP_018331913.1 in cleaving 2-hydroxyisobutyryl-CoA to acetone and formyl-CoA. Interestingly, these crude extracts also show butanone formation from 2-hydroxy-2-methylbutyryl-CoA at nearly the same rate (about 5 nmol min^–1^ mg^–1^), whereas a corresponding cleavage of 2-hydroxy-2-ethylbutyryl-CoA to 3-pentanone was not detected.

## Discussion

In the search for a 2-hydroxyisobutyryl-CoA mutase-independent degradation pathway for short-chain tertiary branched 2-hydroxyacids, we found that the actinobacterial strain *A. chiangmaiensis* DSM 45062 is capable of oxidizing MPD to 2-HIBA and subsequent cleavage to acetone and formic acid. Likely, this cleavage reaction involves the corresponding CoA thioester and proceeds via formyl-CoA formation catalyzed by a novel TPP-dependent 2-hydroxyacyl-CoA lyase.

The strong induction of the lyase-HCL and Mim gene clusters in 2-HIBA-grown cells of strain DSM 45062 clearly indicates that 2-HIBA is initially activated to 2-hydroxyisobutyryl-CoA and that the cleavage product acetone is oxidized via formation of acetol and methylglyoxal, which is a highly toxic ketoaldehyde (Subedi et al., 2008). Efficient removal of the latter is, therefore, of outmost importance and seems to be guaranteed by activities of the aldehyde dehydrogenases MdpC and the acetone-induced WP_18333199.1. Besides, acetol degradation might also involve reduction to propane-1,2-diol and further oxidation to lactaldehyde and lactic acid, as previously suggested for *E. coli* (Subedi et al., 2008). In addition to the Mim cluster-associated alcohol dehydrogenase Adh1 and the two aldehyde dehydrogenases mentioned above, various other dehydrogenases are substantially induced and might be involved in acetol, methylglyoxal and lactaldehyde degradation as well. In contrast to acetone metabolism, no specific enzyme for the hydrolysis of formyl-CoA, the postulated second product of the 2-hydroxyisobutyryl-CoA lyase reaction, could be identified. Likely, it is hydrolyzed to CoA and formic acid by constitutive thioesterases with broad substrate specificity (Figure 4). Finally, the oxidation of formic acid turned out to be clearly dependent on selenium and highly likely involves the selenocysteine-containing membrane-bound FDH1 and cytoplasmic FDH3 identified in strain DSM 45062, although the detected abundance of the corresponding proteins was > 50-fold lower than for the other proteins of the lyase-dependent pathway, for example, the lyase, HCL and the large acetone monooxygenase subunit MimA. This result was equally obtained by two different proteome preparation methods and, therefore, seems not to be caused by inefficient protein extraction. More likely, the observed abundance is already sufficient to remove the formic acid produced during 2-HIBA cleavage, as selenocysteine-containing FDHs can show a > 100-fold increased catalytic efficiency compared to their cysteine counterparts (Axley et al., 1991). The general catalytic superiority of selenium over sulfur and its higher resistance to permanent oxidation (Reich and Hondal, 2016; Maroney and Hondal, 2018) are likely the drivers for the widespread occurrence of selenocysteine-containing enzymes in anaerobic as well as aerobic microorganisms (Peng et al., 2016), despite the requirement for selenium-sequestration and incorporation mechanisms.

During prolonged cultivation of strain DSM 45062 on 2-HIBA under selenium-limiting conditions, we observed a molar ratio of formic acid accumulation versus 2-HIBA consumption of only 0.6. This slightly understoichiometric accumulation might be resulting from partial assimilation of formic acid, possibly via reduction to formaldehyde and lyase-catalyzed acyloin condensation with formyl-CoA to glycolyl-CoA, as has been demonstrated for other 2-hydroxyacyl-CoA lyases (Chou et al., 2019). However, the novel lyase of strain DSM 45062 has not yet been biochemically characterized, due to the low activities obtained in crude extracts of wild type cells and after heterologous expression under the assay conditions applied. Considering the abundance of the enzyme in 2-HIBA-grown cells (Table 1), a specific activity of about 250 nmol min^–1^ mg^–1^ (corresponding to a *k*_*cat*_ of 0.26 s^–1^) can be deduced, indicating that enzyme preparation and assay protocols still need to be optimized. In addition, a significant overexpression in *E. coli* could not be established, likely due to insufficient soluble expression as previously observed for human HACL1 and other 2-hydroxyacyl-CoA lyases from prokaryotic origin (Chou et al., 2019; Burgener et al., 2020). In order to purify the enzyme from strain DSM 45062 for kinetic and structural characterization, production of the recombinant protein has to be improved, for example, by employing an actinobacterial expression system such as *M. smegmatis* (Bashiri and Baker, 2015). Likewise, suitable purification protocols have to be established, as our preliminary attempts to remove elution buffer and to concentrate the lyase after standard immobilized metal ion affinity chromatography resulted in precipitation of the heterologous protein. The thus far characterized 2-hydroxyacyl-CoA lyase from *Rhodospirillales* bacterium URHD0017 (Chou et al., 2019) shows a relatively low *K*_*m*_ value with formyl-CoA (*K*_*m*_ = 200 ± 50 μM) for the synthase reaction with short-chain aldehydes and ketones. However, the latter compounds are poor substrates which leads to low catalytic efficiency values for the production of 2-hydroxyacyl-CoAs. In particular, the *K*_*m*_ value for acetone exceeds 1 M, rating the enzyme rather unsuitable for developing an alternative biotechnological route to the important building block chemical methyl methacrylate via 2-HIBA (Rohwerder and Müller, 2010). Attempts to improve the enzyme by mutating various active site residues, however, did not yet result in enhanced synthesis efficiencies of the *Rhodospirillales* enzyme (Chou et al., 2019). Due to the involvement in a dissimilatory pathway for the degradation of 2-HIBA, we expect that the lyase from DSM 45062 is more specific for 2-hydroxyisobutyryl-CoA. Consequently, it might be a better candidate for the biotechnological production of the methyl methacrylate precursor from acetone and C1 compounds. In addition, 2-hydroxy-2-methylbutyryl-CoA is converted at equal rates, while the C6 acyl moiety of 2-hydroxy-2-ethylbutyryl-CoA seems already too bulky for the active site of the enzyme.

Interestingly, another 2-hydroxyacyl-CoA lyase candidate (IMG locus tag DebiaDRAFT_04574) belonging to the TPP-requiring enzyme superfamily has recently been identified in the deltaproteobacterium *Desulfococcus biacutus* KMRActS (Frey et al., 2018). The 685-aa protein is induced in acetone-grown cells (Gutierrez Acosta et al., 2014) and has been proposed to be involved in the anaerobic degradation of the carbonyl compound via 2-hydroxyisobutyryl-CoA formation (Frey et al., 2018). In strain KMRActS, the latter is isomerized by an RcmAB-like mutase to the common metabolite 3-hydroxybutyryl-CoA. Hence, for this energy efficient pathway, both the lyase and the mutase activities seem to be combined in one strain. Although not closely related to the lyase from DSM 45062 (22% identical residues at 84% query cover) and the other thus far identified 2-hydroxyacyl-CoA lyases (Chou et al., 2019), it is reasonable to assume that the deltaproteobacterial enzyme is likewise using formyl-CoA for the condensation reaction, possibly provided by a CoA-acylating formaldehyde dehydrogenase (Chou et al., 2019) or formyl-CoA transferase reaction (Berthold et al., 2008). However, as this pathway is for acetone and not for 2-HIBA degradation, strain KMRActS does not possess HCL for specifically activating the branched carboxylic acid. In conclusion, bacterial 2-hydroxyacyl-CoA lyases have evolved several times and are involved in pathways for the degradation of short-chain ketones and highly branched compounds, such as acetone and isobutene. In this connection, it is worth mentioning that a conserved lyase-HCL gene cluster is also found in *Pseudonocardia* strains (Figure 1B) isolated from the exoskeleton of fungus-farming leafcutter ants (Sit et al., 2015). In this case, 2-HIBA may be formed in the ants’ nest as a metabolite from isobutene produced in the course of fungal degradation of leucine-rich plant material, as the branched amino acid is usually processed via 3-hydroxyisovaleric acid which is known to be readily converted to isobutene and CO_2_ by eukaryotic mevalonate pyrophosphate decarboxylase (Gogerty and Bobik, 2010). Surprisingly, a lyase-HCL gene cluster variant is also present in isoprene-degrading *Rhodococcus* strains. In the latter, the MPD dehydrogenase MpdB is replaced by an only distantly related alcohol dehydrogenase of the glucose-methanol-choline oxidoreductase family (Figure 1B). Nevertheless, this may be indicative of a second bacterial isoprene oxidation pathway, besides the well-established route via a glutathione adduct (van Hylckama Vlieg et al., 1999; McGenity et al., 2018). In the proposed lyase-dependent route, isoprene would be oxidized as shown for isobutene in Figure 1A via the corresponding 1,2-epoxide and 1,2-diol. Finally, the potential lyase substrate 2-hydroxy-2-methylbut-3-enoyl-CoA could be formed which is likely cleaved to methyl vinyl ketone and formyl-CoA. Another substrate for the lyase may be formed by isoprene reduction to isoamylene (Kronen et al., 2019). The latter alkene could be processed analogous to isobutene and isoprene oxidation to 2-hydroxy-2-methylbutyryl-CoA, which is readily used as substrate by the lyase from strain DSM 45062. Considering that natural isoprene emissions on earth are about equivalent to methane emissions and amount to 500 and 600 Mt year^–1^ (Guenther et al., 2006, 2012), this volatile organic compound may be indeed the main driver for the evolution of actinobacterial 2-hydroxyacyl-CoA lyase.

## Materials and Methods

### Chemicals, Bacterial Strains, and Growth Experiments

Chemicals were purchased from Th. Geyer (Renningen, Germany) at the highest purity available. The lyase 2-hydroxyacyl-CoA substrates tested in this study were synthesized from the free carboxylic acids and CoA via thiophenyl esters (Padmakumar et al., 1993). 2-Hydroxy-2-methylbutyric acid was used as racemic mixture of the (*R*)- and (*S*)-enantiomers. The CoA thioesters were characterized and quantified by HPLC-based methods as previously described (Yaneva et al., 2012). MPD was from Taros Chemicals (Dortmund, Germany). Cultures of *A. chiangmaiensis* DSM 45062 (Jiang et al., 2008) were purchased from the German Collection of Microorganisms and Cell Cultures GmbH (Braunschweig, Germany) and grown at 30°C in a mineral salt medium containing the following (in milligrams L^–1^): NH_4_Cl, 760; KH_2_PO_4_, 680; K_2_HPO_4_, 970; CaCl_2_ × 6 H_2_O, 27; MgSO_4_ × 7 H_2_O, 71.2; FeSO_4_ × 7 H_2_O, 14.94; CuSO_4_ × 5 H_2_O, 0.785; MnSO_4_ × 4 H_2_O, 0.81; ZnSO_4_ × 7 H_2_O, 0.44; Na_2_MoO_4_ × 2 H_2_O, 0.25; CoCl_2_ × 6 H_2_O, 0.4; biotin, 0.02; folic acid, 0.02; pyridoxine-HCl, 0.1; thiamine-HCl, 0.05; riboflavin, 0.05; nicotinic acid, 0.05; DL-Ca-pantothenate, 0.05; *p*-aminobenzoic acid, 0.05; lipoic acid, 0.05, and cobalamin, 0.05; initial pH was 7.5. The medium was supplemented with 200 mg L^–1^ yeast extract and either glucose, 2-HIBA or acetone as main carbon source (500 to 1000 mg L^–1^). The trace elements selenium (Na_2_SeO_3_ × 5 H_2_O at 0.03 mg L^–1^) and tungsten (Na_2_WO_4_ × 2 H_2_O at 0.04 mg L^–1^) were added as indicated. In fed-batch experiments on 2-HIBA or acetone, substrate concentrations were maintained between 200 and 1500 mg L^–1^ (Supplementary Figure S5). After consumption of about 1000 mg L^–1^ of carbon source, the culture was supplemented with another quantum of 100 mg L^–1^ yeast extract. The pH was adjusted manually to values between 6.5 and 7.5 with 10% aqueous solutions of NaOH and sulfuric acid. Data shown on substrate consumption and metabolite formation in DSM 45062 cultures represent mean values and standard deviations from at least five independent experiments. Cultures of *E. coli* BL21 (DE3) (Invitrogen) were grown in lysogeny broth at 30°C.

### Proteome Analysis

Cells from fed-batch cultures growing on either acetone or 2-HIBA as main carbon source in mineral salt medium supplemented with selenium were harvested by centrifugation (10 min, 4°C, 5000 × *g*), washed twice in mineral salt medium and stored at −30°C. Proteome analyses were performed as described previously (Haange et al., 2019) with five replicates for each treatment. Mass spectrometric analysis of eluted peptides was performed on a Q Exactive HF mass spectrometer (Thermo Fisher Scientific, Waltham, MA, United States) coupled with a TriVersa NanoMate (Advion, Ltd., Harlow, United Kingdom) source in LC chip coupling mode. MS data processing was performed using Proteome Discoverer (v.2.2, Thermo Fischer Scientific, Waltham, MA, United States) against the protein-coding sequence of *A. chiangmaiensis* DSM 45062 (from NCBI March 2019) supplemented with protein-coding entries of FdhA1 and FdhA2 (see Figure 2C), in total 5,504 sequence entries. Search settings for Sequest HT search engine were set to trypsin (Full), max. missed cleavage sites: 2, precursor mass tolerance: 10 ppm, fragment mass tolerance: 0.05 Da. Carbamidomethylation of cysteines was specified as a fixed modification and the oxidation of methionine and N-acetylation of the protein N-terminus as a variable modification. Proteins were considered as identified when at least one unique peptide passed the false discovery rate below 0.01. In total, we were able to identify 2,848 protein groups comprised of 20,561 peptides, so the coverage of the *A. chiangmaiensis* DSM 45062 proteome was approximately 52%.

### Cloning and Heterologous Expression

The 2-hydroxyacyl-CoA lyase gene (WP_018331913.1) was cloned into expression vector pASG-IBA43 according to the manufacturer’s protocol (IBA Lifesciences) after amplification using the Q5 High-Fidelity DNA Polymerase Master Mix (New England Biolabs), genomic DNA from strain DSM 45062, forward primer 5′-AGC GGC TCT TCA ATG GCG GAC CGG CAG GAC-3′ and reverse primer 5′-AGC GGC TCT TCT CCC GAT CCC TTC CTG ACG GCG-3′. The PCR conditions were initial denaturation of 3 min at 94°C, 25 cycles of 30 s 94°C, 45 s 60°C and 2 min 72°C, and a final elongation of 5 min at 72°C. Chemically competent cells of strain *E. coli* BL21 (DE3) were transformed with the constructed lyase-bearing vector via heat shock at 42°C. Heterologous expression of the lyase in lysogeny broth supplemented with 100 mg L^–1^ ampicillin was induced at 30°C for 5 to 7 hours by 200 μg L^–1^ anhydrotetracycline after an initial growth at 30°C from an optical density at 550 nm of 0.1 to a value of 0.6. Cells were harvested by centrifugation and mechanically disrupted with glass beads (Yaneva et al., 2012) in phosphate buffer (50 mM potassium phosphate, pH 7.2).

### 2-Hydroxyacyl-CoA Lyase Assay

Rates of enzymatic 2-hydroxyacyl-CoA cleavage were determined in a discontinuous GC-based assay. In the case of crude extracts obtained from DSM 45062 cultures via mechanical disruption (see above), 1 mM MgCl_2_, 200 μM TPP, 312 μM 2-hydroxyisobutyryl-CoA and 0.16 g L^–1^ protein were mixed in phosphate buffer (50 mM potassium phosphate, pH. 7.2) at 30°C and 200-μL samples were taken at several time points within a total incubation of up to 25 min (Figure 5A). The samples were immediately transferred into 10-mL headspace GC vials, closed and incubated for 5 min at 70°C to quench the reaction. Then, ketone formation was analyzed by GC methods as described below. The data shown represent mean values and standard deviations from at least five independent experiments. Likewise, lyase activity in *E. coli* crude extracts obtained after heterologous expression was quantified at 30°C mixing 1 mM MgCl_2_, 200 μM TPP and 0.31 g L^–1^ protein with 430 μM of 2-hydroxyisobutyryl-CoA, racemic 2-hydroxy-2-methylbutyryl-CoA or 2-hydroxy-2-ethylbutyryl-CoA. As control, extracts from non-induced cultures as well as from the strain without the lyase expression plasmid were tested for lyase activity against 2-hydroxyisobutyryl-CoA. The detection limit for the lyase assay amounts to an enzymatic ketone (acetone, 2-butanone, or 3-pentanone) formation of 0.1 nmol min^–1^ mg^–1^.

### Analytics

In DSM 45062 cultures, consumption of substrates and formation of metabolites were routinely monitored by HPLC (Shimadzu Corporation) equipped with a Hi-Plex H column (300 mm × 7.7 mm; Agilent Technologies) and refractive index detector (Becher et al., 2018). In addition, acetone was quantified by headspace GC (Agilent Technologies) employing an Optima Delta-3 column (60 m × 0.32 mm × 0.25 μm; Macherey-Nagel) and flame ionization detection (Becher et al., 2018). Formation of ketones in bacterial cultures and in the lyase assays was verified by a GC system (Agilent Technologies) equipped with an Optima Delta-3 column (30 m × 0.25 mm × 0.25 μm; Macherey-Nagel) and mass spectrometer (Becher et al., 2018). The resulting mass spectra were compared with spectra from pure standard chemicals as well as with most-probable matches by the National Institute of Standards and Technology library database. Proteins were quantified using the Bradford reagent from Merck (Darmstadt, Germany).

### Bioinformatics

Similarity of proteins was determined by BLAST (Altschul et al., 1997) and sequences were aligned by Clustal Omega (Sievers and Higgins, 2018). For identifying the selenocysteine insertion sequence (SECIS) element accompanying the selenocysteine UGA codon in FdhA subunit genes (Supplementary Figure S2), the bSECISearch tool^1^ was used (Zhang and Gladyshev, 2005). Secondary RNA structures were drawn with Forna^2^ (Kerpedjiev et al., 2015). Sequences of FDH subunits and other predicted proteins with ambiguous annotation were searched for functional sites with the NCBI Conserved Domain tool (Marchler-Bauer et al., 2017) and by InterPro^3^ (Finn et al., 2017).

## Data Availability Statement

The mass spectrometry proteomics data have been deposited to the ProteomeXchange Consortium via the PRIDE partner repository (Vizcaino et al., 2014; Perez-Riverol et al., 2019) with the dataset identifier PXD017800.

## Author Contributions

TR, M-TR, and JP conceived and directed the project. M-TR, NJ, and JP performed the experiments and all the authors were involved in data analysis as well as method design. TR wrote the manuscript with substantial input from all the authors.

## Conflict of Interest

The authors declare that the research was conducted in the absence of any commercial or financial relationships that could be construed as a potential conflict of interest.

## Footnotes

1 http://genomics.unl.edu/bSECISearch/

2 http://rna.tbi.univie.ac.at/forna/

3 https://www.ebi.ac.uk/interpro/search/sequence/
