# Supplementary material for: Actinobacterial Degradation of 2-Hydroxyisobutyric Acid Proceeds via Acetone and Formyl-CoA by Employing a Thiamine-Dependent Lyase Reaction
Source: Front Microbiol. 2020 Apr 15;11:691. doi: 10.3389/fmicb.2020.00691 (PMC7176365; doi:10.3389/fmicb.2020.00691)
Supplement: Supplementary file 1 [file Data_Sheet_1.DOCX]

Supplementary Material

# Morphology of strain *Actinomycetospora chiangmaiensis* DSM 45062 in liquid cultures

**Supplementary Figure S1.** The actinobacterial strain *Actinomycetospora chiangmaiensis* DSM 45062 does not grow as planktonic cells but exclusively forms cell aggregates of up to 3 mm in diameter in liquid cultures. For visualization, biomass was collected by filtration (0.2 µm pore size) after fed-batch growth on 2-HIBA as main carbon source in mineral salt medium supplemented with selenium.

# Additional information on formate dehydrogenases in strain *Actinomycetospora chiangmaiensis* DSM 45062

**Supplementary Figure S2.** Gene clusters in *Actinomycetospora chiangmaiensis* DSM 45062 encoding selenocysteine-dependent formate dehydrogenases. SECIS elements of the FdhA subunit genes were predicted by the bSECISearch tool (http://genomics.unl.edu/bSECISearch/). Alignment shows residues of the conserved domain involved in binding to Mo/W in formate dehydrogenases. FDH-N_A, *E. coli* FDH-N alpha subunit (UniProt ID P24183); FDH-O_A, *E. coli* FDH-O alpha subunit (UniProt ID P32176); FDH-H_A, *E. coli* FDH-H alpha subunit (UniProt ID P07658); FDH1_A, *Methylorubrum extorquens* AM1 formate dehydrogenase 1 alpha subunit (NCBI ID ACS42636.1); FdsA, *Cupriavidus necator* H16 formate dehydrogenase alpha subunit (NCBI ID WP_011614623.1).

**Supplementary Figure S3.** Comparison of FDH1 from *Actinomycetospora chiangmaiensis* DSM 45062 with the formate dehydrogenase FDH-N from *E. coli*.

**Alpha subunit.** The predicted 1078-aa subunit FdhA1 (currently not correctly annotated at NCBI) is homologous to the 1015-aa *E. coli* FDH-N alpha subunit (UniProt ID P24183) sharing 37% identical residues at 93% query cover, including the catalytic site selenocysteine U190 residue. FdhA1 is predicted to contain a bis-metallopterin guanine dinucleotide cofactor, likely with a molybdenum(VI) metal center (Mo). Thus, electrons would be channeled from formate through the Mo-containing cofactor and iron-sulfur clusters ([FeS]) to the beta subunit.

**Beta subunit.** FdhB1 (NCBI ID WP_018332710.1) shares 39% identical residues at 89% coverage with the *E. coli* FDH-N beta subunit (UniProt ID P0AAJ3) transferring the electrons further to the gamma subunit.

**Gamma subunit/FdhC1.** The genome of strain DSM 45062 does not harbor any genes encoding FDH-N gamma subunit homologs, which contain characteristic heme b-type redox centers (heme b). However, the third ORF in the FDH1 gene cluster is predicted to encode the 366-aa transmembrane protein FdhC1 (NCBI ID WP_018332711.1) which is partially related (24 to 26% identities at 55 to 56% query cover) to nitrite reductase pathway protein D from *E. coli* (NrfD, UniProt ID P32709) and polysulfide reductase subunit C from *Wolinella succinogenes* (PsrC, UniProt ID P31077). Therefore, it is speculated here that FdhC1 functions as the gamma subunit of FDH1, likely transporting the electrons to the quinone pool (Q).

**Possible formate exporter WP_084681825.1.** The fourth ORF in the gene cluster is predicted to encode a transmembrane permease protein. Although substrate specificity of related proteins (DUF318 family proteins, Pfam entry PF03773) is not available, the presence in the FDH1 gene cluster might, nevertheless, be indicative of a role in formate transport.

**Supplementary Figure S4.** Comparison of FDH3 from *Actinomycetospora chiangmaiensis* DSM 45062 with formate dehydrogenases from *Methylorubrum extorquens* AM1 and *Cupriavidus necator* H16.

**Alpha subunit.** The predicted 640-aa subunit FdhA3 (NCBI ID WP_084681743.1) is homologous to the metallopterin-binding C-terminal domain of formate dehydrogenase alpha subunits from strains AM1 (NCBI ID ACS42636.1) and H16 (NCBI ID WP_011614623.1) showing identities of 37 and 36% at 96 and 97% coverage, respectively. In these cytoplasmic NAD^+^-reducing formate dehydrogenases, the active site selenocysteine of FdhA3 (U80) is not conserved. The AM1 alpha subunit contains a tungsten (W) instead of a molybdenum metal center.

**Gamma subunit.** While electron transfer from the metallopterin cofactor to the next subunit proceeds through the N-terminal iron-sulfur cluster domain of AM1 and H16 alpha subunits, FDH3 is predicted to possess a separate but homologous 316-aa protein FdhC3 (NCBI ID WP_018332444.1) for this function.

**Beta subunit.** The N- and C-terminal domains of the 614-aa FdhB3 (NCBI ID WP_018332443.1) resemble the NuoE/Nqo1 and NuoF/Nqo2 subunits, respectively, of the mitochondrial respiratory chain complex I and are also homologous to the iron-sulfur cluster and flavin mononucleotide (FMN) domains of the AM1 beta (35% identical residues at 93% query cover) and H16 gamma plus beta subunits (34 to 48% identical residues, respectively). Hence, electrons from FdhC3 are likely transferred via the various redox centers of FdhB3 to NAD^+^.

# Feeding regime in fed-batch cultures of *Actinomycetospora chiangmaiensis* DSM 45062

**Supplementary Figure S5.** Fed-batch cultivation of strain *Actinomycetospora chiangmaiensis* DSM 45062 on 2-HIBA as main carbon source in mineral salt medium supplemented with selenium. (A) Course of 2-HIBA concentrations and cumulative consumption in a representative culture incubated over 27 days. The initial 2-HIBA concentration amounted to 1000 mg L^-1^. On days 15 and 22, the culture was supplemented with about 1000 mg L^-1^ 2-HIBA in order to maintain substrate concentrations above 200 mg L^-1^. (B) Cumulative 2-HIBA consumption as mean value and standard deviation of five independent experiments.
